# Network Analysis of Swine Shipments in China: The First Step to Inform Disease Surveillance and Risk Mitigation Strategies

**DOI:** 10.3389/fvets.2020.00189

**Published:** 2020-04-28

**Authors:** Kathleen O'Hara, Rui Zhang, Yong-Sam Jung, Xiaobing Zhou, Yingjuan Qian, Beatriz Martínez-López

**Affiliations:** ^1^Center for Animal Disease Modeling and Surveillance, School of Veterinary Medicine, University of California, Davis, Davis, CA, United States; ^2^MOE Joint International Research Laboratory of Animal Health and Food Safety, College of Veterinary Medicine, Nanjing Agricultural University, Nanjing, China; ^3^Bright Food (Group) Co., Ltd., Shanghai, China

**Keywords:** social network analysis, pig movements, disease spread, risk-based surveillance, swine diseases

## Abstract

China's pork industry has been dramatically changing in the last few years. Pork imports are increasing, and small-scale farms are being consolidated into large-scale multi-site facilities. These industry changes increase the need for traceability and science-based decisions around disease monitoring, surveillance, risk mitigation, and outbreak response. This study evaluated the network structure and dynamics of a typical large-scale multi-site swine facility in China, as well as the implications for disease spread using network-based metrics. Forward reachability paths were used to demonstrate the extent of epidemic spread under variable site and temporal disease introductions. Swine movements were found to be seasonal, with more movements at the beginning of the year, and fewer movements of larger pigs later in the year. The network was highly egocentric, with those farms within the evaluated production system demonstrating high connectivity. Those farms which would contribute the highest epidemic potential were identified. Among these, different farms contributed to higher expected epidemic spread at different times of the year. Using these approaches, increased availability of swine movement networks in China could help to identify priority locations for surveillance and risk mitigation for both endemic problems and transboundary diseases such as the recently introduced, and rapidly spreading, African swine fever virus.

## Introduction

The ongoing expansion of national and international trade has increased the movement of animals, products, and disease globally. China's pork industry has been dramatically impacted by these market changes, with import tonnage quadrupling between 2005 and 2015 (1). The swine industry in China is also seeing an increasing consolidation of traditional farms of <50 animals, into large-scale facilities supporting thousands of pigs (1). The implications of these changes include: increased pig density and trade, higher risk of introduction of novel pathogens, faster disease spread, higher disease incidence, and the generation of novel pathogen strains (2–5). This has been evident with the arrival of African Swine Fever virus (ASF) in August 2018, as well as the dramatic impact that other swine diseases such as PRRS or PED are having in the country (6). These changes in swine production, as well as the introduction of ASF, have increased the necessity for traceability and efficient data-driven methods to support disease surveillance, prevention, and outbreak response.

Understanding the social network of a production system allows for risk assessment of disease dissemination within the local industry (7), as well as a faster response in case of an epidemic (8). Large swine operations are highly integrated and multi-site—commercial swine production systems use separate sites for sow farms, gilt development, nursing, finishing farms, boar studs, and culling, requiring the frequent shipment of live pigs from location to location (9). These intrinsic pig movement requirements provide opportunities for disease introduction and spread. Understanding when and where these points of contact occur, and the network structure and vulnerabilities, may help to strategically allocate risk-based, more cost-effective, preventive and control measures.

Social network analysis (SNA) has been demonstrated to be a valuable tool to describe these pig movement network structures (10). It has been used to evaluate the movement network dynamics and helps to quickly identify the individual farms, areas and time periods that may pose the highest risk for disease introduction to the system (11–14). These insights allow for implementation of risk mitigation strategies at these spatial or temporal hotspots (14), as well as more realistic disease modeling.

Understanding the social structure of a typical swine facility in China is a first step toward risk analysis in the Chinese swine industry; however, there is currently very limited published information about Chinese swine farm demographics, or the trade and contact patterns at fine spatio-temporal scale (daily/weekly and at farm level). This lack of information is a critical gap in China's animal health and emergency response plans. Moreover, the increasing demand for animal protein due to population growth and westernization of the diet, makes food safety and security a primary concern for China (15). Limiting disease outbreaks, especially those that may severely impact food production and international trade (such as the current ASF epidemic) is critical if China is to meet these consumer demands and maintain industry health standards. SNA applied to the swine industry may also allow for the identification of potential superspreaders or super-receivers of disease within China's pork industry chain, providing targeted locations for surveillance and risk mitigation (13). Food security programs in China should prioritize characterization of the swine trade network as the first step in risk assessment.

We undertook this study as a first exploration of the social network of the pig trade in China.

Our primary objectives were: to describe the network structure in a typical multi-site system in China; to describe pig movement spatio-temporal dynamics; and to identify priority farms that may contribute to the risk of disease introduction and spread. The increased availability of swine demographics and trade data in China will help to better understand, and even predict, disease transmission patterns, which will support risk-based surveillance and control strategies for both endemic and emerging swine diseases such as African Swine fever.

## Materials/Methods

### Data

Live pig movement data was collected from a large-scale multi-site pig producer in China. These data were translated from Mandarin to English for analysis. Farms were anonymized to protect producer and citizen consumer privacy. The final dataset included movement information to/from the farms belonging to the participant multi-site pig production system between Jan 1 and Dec 31, 2017. Shipment information included: origin, destination, date, type of shipments (culling or finisher), type of pig, total weight, number of pigs, average weight per pig, and distance moved. Pigs types were classified as: boar, commercial pig, feeder, grower, sow, or unspecified. A total of 2,567 shipments were considered in this study. Data were collected, validated and cleaned in Microsoft Excel 2016 and R language (v.3.4.1) (16, 17).

### General Approach

In a first step, we constructed and evaluated the characteristics and properties of the complete network (for the whole study period). In a second step, we generated and evaluated the properties of the dynamic network (i.e., considering the complex dynamics of the edge formation and dissolution over time). Finally, we used the dynamic network to evaluate the potential disease transmission and epidemic sizes over the network, considering the actual farm-to-farm contacts under diverse epidemiological scenarios and computing the forward reachability paths for all nodes in the network.

### Static Network Analysis

The complete “static” network for the whole study period (i.e., 1 year) was defined using swine production sites as nodes or vertices, and shipments of live pigs as edges. After the complete static network was generated, we then focused on the farm-to-farm (“to live”) movements (i.e., subset of the network removing movements to slaughterhouses). The static networks studied were treated as non-weighted. Network parameters including number of nodes, number of edges, diameter, edge density, average path length, and transitivity were calculated to study the properties and characteristics of the network. Centrality measures of in-degree and out-degree were calculated for each node. Briefly, in-degree is defined as the number of incoming shipments to a farm, out-degree is the number of outgoing shipments from a farm (18, 19). Diameter is the longest of all the shortest path lengths between nodes in the network (18, 19). Edge density is the ratio of the number of edges observed in the network to the number of possible edges (18, 19). Average path length is the mean length of all the shortest paths between nodes in the network (20). Transitivity coefficient is the sum of the proportion of nodes that are connected to other nodes; this parameter is also known as the clustering coefficient (18, 20). The igraph package [v 1.1.2; (21)] in R Studio [v 3.4.1; (17)] was used to generate and describe the static network and evaluate network parameters. Edge density, diameter, average path length, and transitivity were calculated under the igraph package using functions: edge_density, diameter, mean_distance, and transitivity, respectively. Type global was used for the transitivity function. The visNetwork package was also used for network visualization (22).

### Network Dynamics and Epidemic Size

The network Dynamic (23), ndtv (24), and tsna (25) packages within R were used to construct and evaluate the dynamic properties and characteristics of the complete and farm-to-farm pig movement networks, and then to estimate the epidemic size within the resultant dynamic network.

We computed the forward reachability path, which has been previously shown to be a good predictor of epidemic size (11, 26), for each node in the complete dynamic network using the tPath function. Forward reachability is defined as the extent to which an introduced infection can spread through the network—given an introduction at farm A, based on the network structure or contacts, to which farms would an infection be expected to move (11). For each initial vertex in a directed network, tpath searches out the sequence and distance of vertices that are reachable following paths constrained by edge timing. Results were presented using graphs, transmission timelines and video (Supplementary Video 1).

## Results

**Figure 1:**
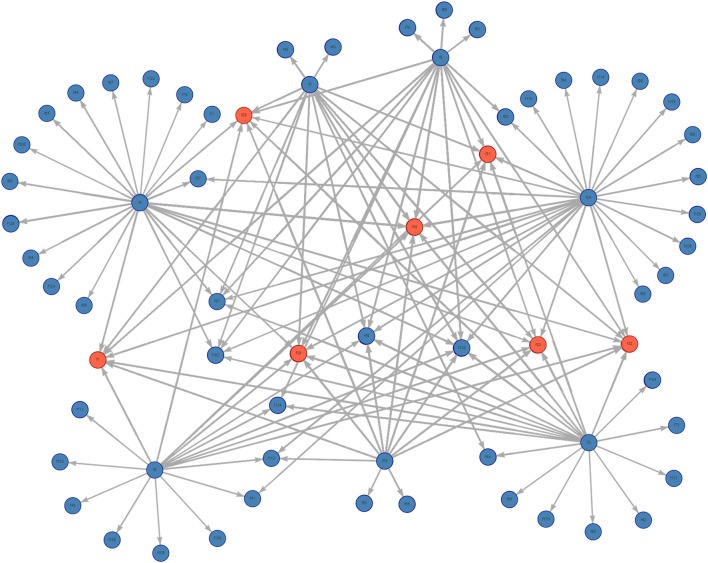
Graph of the complete pig movement network for the studied Chinese production system. Farms are in blue, slaughterhouses are in red.

**Figure 2:**
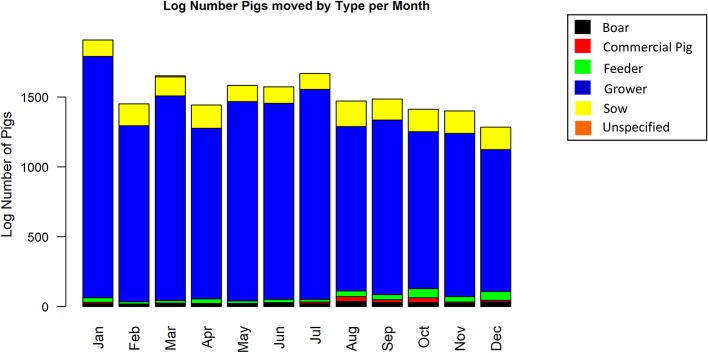
Log number of shipments by type of pig and month of the year.

**Figure 3:**
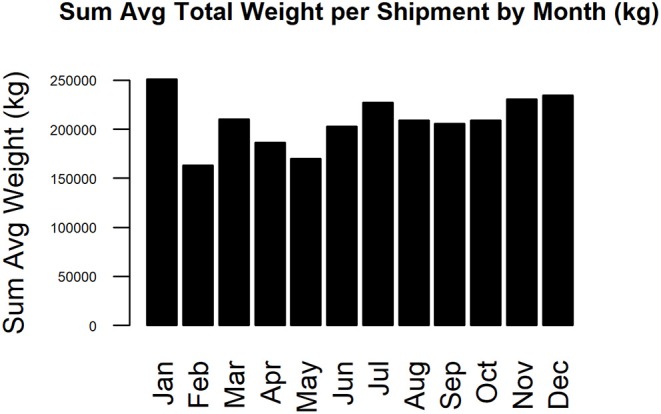
Sum average weight per shipment by month (kg).

The complete static network including all movements during 2017 for our studied Chinese production system contained 67 nodes and 2,567 edges (Figure 1). The network shipments displayed seasonality, with the overall number of shipments being highest in January, then declining throughout the year (Figure 2). Growers were consistently the predominant type of pig being shipped, with sows being the next most common. The highest sum average weight was observed in January, with a drop off in February and May, then a gradual increase through the rest of the year (Figure 3).

The subsetted farm-to-farm network, discarding movements to slaughter, had 50 nodes and 485 edges. The characteristics of the complete vs. farm-to-farm networks are outlined in Table 1. Both networks conform a weakly-connected component, in which all farms are connected by some path (ignoring direction) and are characterized by short diameters and path lengths. The transitivity, or clustering coefficient, for both networks was zero. The farm-to-farm network shows low edge density at 0.198. Within our farm-to-farm network we identified those farms with the highest in-degree and highest out-degree. Those with the highest out-degree—our potential super-spreaders—included farms f24, f4, and f3 with a total of 164, 86, and 65 outgoing movements, respectively. Those with the highest in-degree—our potential super-receivers—included farms f132, f97, and f70 with a total of 260, 38, and 28 incoming movements, respectively.

**Table 1:** Network parameters for the complete and subsetted farm-to-farm networks.

**Global parameters**	**Complete network**	**Farm-to-farm network**
Nodes	67	50
Edges	2,567	485
Edge density	0.5805	0.198
Diameter	1	1
Average path length	1	1
Transitivity	0	0
**Node parameters**	**Mean (Min, Max)**	**Mean (Min, Max)**
In-degree	38.3134 (0, 525)	9.7 (0, 260)
Out-degree	38.3134 (0, 667)	9.7 (0, 164)

**Figure 4:**
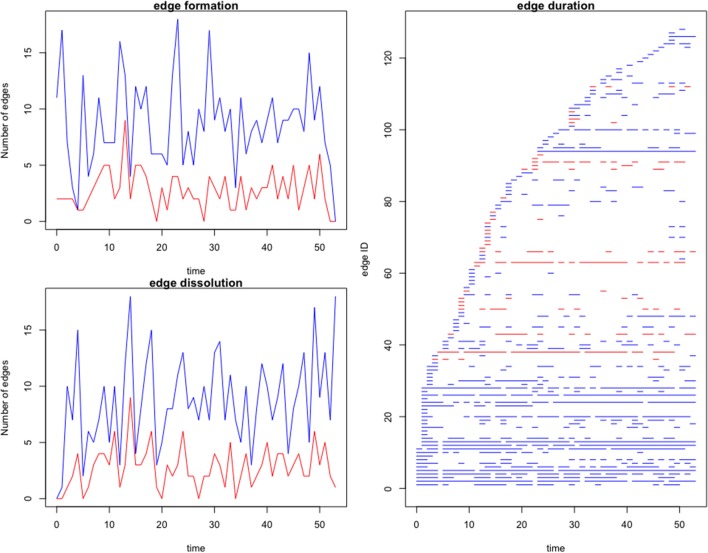
Edge formation and dissolution rates, and edge duration per node of the complete (blue) and farm-to-farm (red) pig movement network for the studied Chinese production system. Time is in weeks. Edge formation is the generation of a contact between two farms. Edge dissolution is the discontinuation of contact between two farms. The edge duration is the length of time (in weeks) that two given nodes had ongoing contact, or shipments between them.

A dynamic network was evaluated for both the complete and farm-to-farm networks. Edge formation, dissolution and duration are graphed and compared in Figure 4. We observe a high rate of connectivity and duration among a few core farms, which compose a stable community evident in our edge statistics and visualized on the dynamic network movie (Supplementary Video 1). The remaining edges are sporadic and short-lived. The complete network, including movements to slaughter, displayed higher rates of edge formation and edge duration, than the farm-to-farm network (Figure 4).

**Figure 5:**
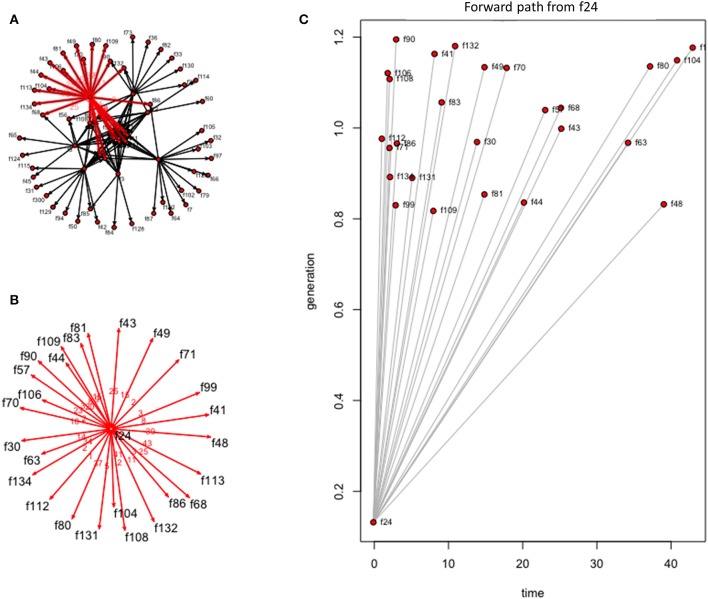
Forward reachability path for farm 24 of the dynamic farm-to-farm network for the studied Chinese production system and considering the entire study period (i.e., 53 weeks). **(A)** The forward reachability within the farm-to-farm network when an infection is started at farm 24. **(B)** Visualization of those farms that could be contacted, and thus become infected, given a disease introduction at farm 24. **(C)** Forward reachability for farm 24 plotted by time in weeks.

**Figure 6:**
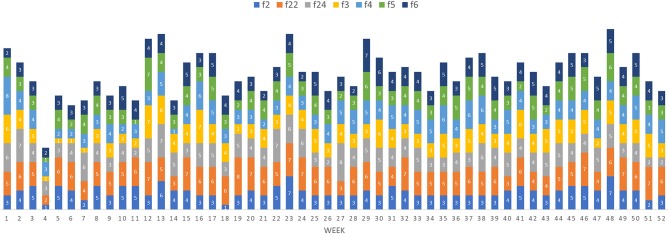
Potential maximum epidemic size (i.e., number of farms infected) based on the forward reachability path for different index cases (farm 2, 22, 24, 3, 4, 5, and 6) per week when the disease is theoretically initiated at the beginning of the week.

In evaluating how a disease would move through this network following introduction, we used forward reachability paths. We illustrated the maximum forward reachability path of f24 in Figure 5, highlighting the extent and locations where disease would likely disseminate to, given a disease introduction at this farm and assuming disease may disseminate during the whole study period. The forward reachability paths identified farms f24, f4, f2 as the potential main superspreaders when considering the whole study period. Farms f24, f4, and f2 could potentially infect 40, 34, and 31% of the farms within our production system, respectively. Farms f24 and f4 would have more contribution to disease spread if infected early in the year, while f5 and f2 would contribute to more disease spread later in the year (Table 2; Figure 6). The potential epidemic size, and the farms that would contribute most to disease spread, change throughout the year (Figure 6).

**Table 2:** Potential maximum epidemic size (i.e., number of farms infected) based on the forward reachability path for different index cases and time periods when the disease is theoretically initiated (in weeks).

**ID**	**Index farm**	**t1-10**	**t10-20**	**t20-30**	**t30-40**	**t40-50**	**t1-53**
1	f2	10	11	9	10	12	21
2	f22	8	8	11	8	10	13
3	f24	13	14	12	11	11	27
4	f3	9	11	9	11	10	15
5	f4	13	11	8	7	5	23
6	f5	7	10	10	12	13	17
7	f6	3	9	10	9	10	19

*Cell colors have increasing scale from light yellow to red and are proportional to the number of farms infected. The maximum epidemic size considering the whole study period is shown in the t1-53 gray column*.

## Discussion

This study analyzed the pig movement network structure and characteristics of a typical multisite swine production system in China and used forward reachability paths to illustrate the potential spread of swine diseases under diverse epidemiological scenarios (different index cases and time of infections). Despite having only 1 year of information, data suggest that the system is seasonal and predominated by the movement of growers. This seasonality likely contributes to the variable impact certain farms had on epidemic spread at different times of the year, and may be explained in part by the timing of Chinese traditional festivals. Drops in epidemic size correspond to multiple of these events: Chinese New Year (Jan 28; week 4), Lantern Festival (Feb 11; week 6), Qingming (Apr 4, week 14), Dragon Boat (May 30, week 22), Mid-autumn (Oct 4, week 40), and Double Ninth (Oct 28, week 43). Presumably more people are away from work, limiting swine shipments, and thus disease spread during these periods.

The farm-to-farm network consisted of one giant weakly-connected component, with a clustering coefficient of zero or no clustering observed. The lack of clustering and the predominance of stars structures in the network are typical in egocentric networks, and in this case is attributable to the single source of the data (i.e., information from one production system, with no information on the external farms contacting our production system). The lack of additional outgoing movement information from what appear to be isolated receiving farms (those with limited movements, and short edge duration), prevents us from getting a better idea of their interconnectivity with this and/or other production systems.

Node centrality and reachability parameters allowed us to identify potential farms that can act as super-spreaders or super-receivers within the network. Super-spreaders, those farms with high out-degree and highest reachability, represent those most likely to have a key role in the dissemination of disease within the network. Super-receivers, those farms with high in-degree, represent those most likely to introduce diseased animals to their farm, and thus become infected. The most cost-effective preventive and control measures will be those targeting farms characterized as super-spreaders for the implementation of risk-mitigation strategies (i.e., biosecurity, vaccination, quarantine etc.), while our super-receivers should be prioritized for enhanced surveillance programs.

Within this study, we evaluated both the complete network and the farm-to-farm network that excluded movements to slaughter. For most infectious diseases, slaughterhouses act as dead ends. In terms of assessing disease spread through a system, it would therefore make sense to focus on those movements that went on to live. However, given the high level of concern for spread of ASF via fomites and contaminated meat products, infected animals that reach slaughterhouses and are not identified, and other pigs who many become infected while awaiting slaughter, pose a dissemination risk if their tissues reach the market (27).

This model could be improved with the addition of movement data for other swine production systems, and better information about node parameters such as farm size, production type, disease status, or biosecurity and management practices, which was not available for the current study. The use of forward reachability paths was considered a fast and reliable approach to estimate the epidemic size for all the nodes and different time periods in the study (11, 28, 29). This approach has been proved to be a good substitute for SIR modeling techniques, particularly in situations like this, where there are low number of secondary contacts due to the egocentric nature of the network. Overall, our approach provides an adequate platform from which to understand how disease could move through the studied Chinese production system.

Using social network analysis of a representative, large multi-site swine production system in China, we were able to identify target farms for risk-based surveillance and disease mitigation efforts. This approach allows us to inform on the most effective and cost-efficient approaches to risk mitigation, disease management, and outbreak response in this particular production system, and can be easily adapted to other production systems if data becomes available. Future directions should include the incorporation of more data about on-farm demographics, farm type and management practices within our network, as well as the incorporation of additional production systems. Creating a spatially explicit network and incorporating the distance between farms and the transportation routes would allow the inclusion and evaluation of other transmission pathways (i.e., airborne transmission, truck movements, fomites, etc.) for stronger predictions of disease dissemination, particularly in those areas with high pig farm density. Additionally, the model could be used to evaluate the efficacy of specific biosecurity or riskbased interventions.

To the authors' best knowledge this study represents the first description of the pig movement patterns in a large-scale Chinese swine production system. We have provided an initial exploration of the swine movement patterns in this system and have demonstrated how the use of social network analyses can be used to inform surveillance and risk mitigation strategies to improve decision-making, and disease prevention and control, within the Chinese swine industry. Given that China has just experienced its first incursion of ASF, the availability of more swine trade data could help to better understand ASF transmission dynamics, as well as to prevent and control further outbreaks. By better understanding the contacts and movement structure of the Chinese pork system, resources may be more efficiently targeted at priority locations for more timely disease mitigation. We recommend the expansion and utilization of this approach as a benchmark in the food safety and emergency response plan for China's swine industry moving forward.

## Data Availability Statement

The datasets generated for this study are available on request to the corresponding author.

## Author Contributions

KO'H performed the data curation and validation, development of the Rcode, and formal analysis under supervision of BM-L and YQ. YQ initiated recruitment of the farms included in this study, and collected, organized, and translated the raw data in collaboration with RZ, Y-SJ, and XZ, as well as contributed to critical interpretation of results and implications for the Chinese swine industry. BM-L contributed to the development of the R-code with KO'H, as well as supervised the development, implementation and interpretation of the analytic approach, model parameterization and model diagnostics. KO'H performed the initial draft preparation. All authors contributed to the project conceptualization and critical review and editing of the submitted manuscript and extensively reviewed and edited the manuscript before submission.

## Conflict of Interest

XZ was employed by the company Bright Food (Group) Co., Ltd. The remaining authors declare that the research was conducted in the absence of any commercial or financial relationships that could be construed as a potential conflict of interest.

## Supplementary Material

Supplementary Video 1 Visualization of the dynamic farm-to-farm network movements across time. Farms with the highest number of movements of sows in red, the remaining farms in blue. Node size is proportional to the cumulative number of movements in the entire time period: small <50, medium 50–200, large >200. Time is presented in weeks.
